# Fluoride Fiber-Based Plasmonic Biosensor with Two-Dimensional Material Heterostructures: Enhancement of Overall Figure-of-Merit via Optimization of Radiation Damping in Near Infrared Region

**DOI:** 10.3390/ma12091542

**Published:** 2019-05-10

**Authors:** Anuj K. Sharma, Ankit Kumar Pandey, Baljinder Kaur

**Affiliations:** Physics Division, Department of Applied Sciences, National Institute of Technology Delhi, Narela, Delhi 110040, India; ankit.pandey@nitdelhi.ac.in (A.K.P.); baljinderkaur@nitdelhi.ac.in (B.K.)

**Keywords:** 2D materials, heterostructure, plasmon, sensor, figure of merit, infrared, fluoride fiber

## Abstract

Two-dimensional (2D) heterostructure materials show captivating properties for application in surface plasmon resonance (SPR) sensors. A fluoride fiber-based SPR sensor is proposed and simulated with the inclusion of a 2D heterostructure as the analyte interacting layer. The monolayers of two 2D heterostructures (BlueP/MoS_2_ and BlueP/WS_2_, respectively) are considered in near infrared (NIR). In NIR, an HBL (62HfF_4_-33BaF_2_-5LaF_3_) fluoride glass core and NaF clad are considered. The emphasis is placed on figure of merit (FOM) enhancement via optimization of radiation damping through simultaneous tuning of Ag thickness (d_m_) and NIR wavelength (λ) at the Ag-2D heterostructure–analyte interfaces. Field distribution analysis is performed in order to understand the interaction of NIR signal with analyte at optimum radiation damping (ORD) condition. While the ORD leads to significantly larger FOM for both, the BlueP/MoS_2_ (FOM = 19179.69 RIU^−1^ (RIU: refractive index unit) at d_m_ = 38.2 nm and λ = 813.4 nm)-based sensor shows massively larger FOM compared with the BlueP/WS_2_ (FOM = 7371.30 RIU^−1^ at d_m_ = 38.2 nm and λ = 811.2 nm)-based sensor. The overall sensing performance was more methodically evaluated in terms of the low degree of photodamage of the analyte, low signal scattering, high power loss, and large field variation. The BlueP/MoS_2_-based fiber SPR sensor under ORD conditions opens up new paths for biosensing with highly enhanced overall performance.

## 1. Introduction

The advancements in the field of two dimensional (2D) materials have opened up new paths for their application in various optoelectronics devices [1,2]. Remarkable developments in micromechanical exfoliation techniques have introduced high-quality 2D organic and inorganic materials [3]. Graphene, a monolayer counterpart of graphite, has found a large number of applications in optical devices like detectors and sensors [4,5,6]. Other 2D materials, such as transition metal chalcogenides (TMDs), stanene, germanene, phosphorene, etc., have also been utilized in several photonic applications [1,7,8]. Optical sensors based on surface plasmon resonance (SPR) have obtained widespread popularity because of their fast response, accuracy, and ease of fabrication. P-polarized waves, which exist at metal-dielectric interface (MDI), are referred to as surface plasmon polaritons (SPPs), and their resonant oscillation under the influence of an external field is known as SPR.

Discussing SPR sensors and 2D materials together, it can be observed that multilayer graphene has been employed in SPR-based sensors for sensitivity enhancement due to its increased surface adsorption [9,10,11,12]. However, increasing the number of graphene layers causes an increase in the damping within the layers, which affects the sensing precision [13]. In this context, the combination of graphene with other 2D materials, forming a heterostructure, has recently been explored for sensitivity enhancement [14,15,16]. The favorable optical properties of heterostructures based on other 2D materials, e.g., Blue phosphorene (BlueP), have received application in SPR sensors founded on prism-based Kretschmann-Raether (KR) configurations [17]. However, the KR configuration is bulky in nature. As a feasible solution, fiber-optic SPR sensors are preferred, owing to their miniaturized and flexible design, which is suitable for remote sensing applications with easy integration [18,19]. In recent years, several experimental and theoretical research works have been reported on fiber SPR sensors with inclusion of 2D material coating [20,21]. Recently, several research works have been reported based on the application of 2D material (graphene and MoS_2_)-assisted heterojunction in fiber SPR sensors for figure of merit enhancement by tuning the radiation damping (RD) [22,23]. 

Heterostructures of BlueP and TMDs are potential materials to be explored for SPR-based biosensing applications. The heterostructure can be formed easily between BlueP and TMDs, as both possess hexagonal lattice structure [24]. The van der Waals (vdW) force of attraction is responsible for holding both the monolayers. This heterostructure formation will increase the stability of BlueP from external agents. The heterostructure contact with analyte leads towards field enhancement at the interface [7] and increased light absorption [25] owing to vdW force of attraction. 

The present work is focused on the simulation and analysis of fluoride fiber-based SPR biosensors with monolayer BlueP/TMDs (TMD: WS_2_ or MoS_2_) heterostructures for the detection of malignancy in liver tissues in the near infrared (NIR) region. The novelty of the present work lies in the inclusion of BlueP/TMDs with fluoride fiber for sensing applications and the introduction of a new performance parameter, i.e., combined figure of merit (C-FOM) for overall performance analysis. To the best of our knowledge, this is the first work based on the application of monolayer 2D heterostructures (BlueP/TMDs) in fiber-optic SPR sensors for biosample detection. The study is conceptualized for the purpose of enhancing sensing performance by tuning the radiation damping at the metal–2D heterostructure–analyte interfaces. To this end, a detailed analysis is carried out leading to simultaneous optimization of silver (Ag) layer thickness and NIR wavelength for sensing performance enhancement. The field enhancement under resonance conditions is also demonstrated. The sensor’s performance is more comprehensively evaluated in terms of its low photodamage, low signal scattering, high power loss, and large field variation.

## 2. Theory and Design Consideration

**Figure 1:**
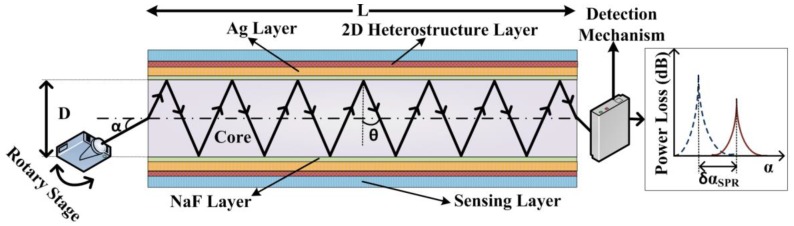
Schematic diagram of the proposed multilayered FOSPR sensor with output power loss spectra. L is the sensing length of fiber, D is the fiber core diameter. The BlueP/TMD layer acts as the analyte interacting layer.

A five-layer structure consisting of fiber core, clad medium, Ag layer, BlueP/TMD (WS_2_ or MoS_2_) heterostructure, and analyte is considered. Multimode heavy metal-doped fluoride optical fiber (HBL: 62HfF_4_-33BaF_2_-5LaF_3_) with core diameter (D) of 400 µm is considered (shown in Figure 1). Several variants of fluoride fiber, including ZBLAN (55.8ZrF4-14.4BaF2-5.2LaF2-3.8AlF3-20.2NaF), ZBLA (57ZrF4 -36BaF2-4AlF3-3LaF3), Aluminium fluoride (AlF_3_) and Indium fluoride (InF), are commercially available [26] that can be considered for possible experimental realization of the proposed sensor design. The clad medium should be selected carefully in order to have a sufficiently large range of incident angle (α), keeping in mind the maximum permissible value, $\alpha_{m}=sin^{-1}\left(\sqrt{n_{core}^{2}-n_{clad}^{2}}\right)$, where $n_{core}$ and $n_{clad}$ represent the refractive index (RI) of core and clad materials, respectively. For the above-mentioned purpose, an NaF layer with a thickness of 5 nm was chosen as clad medium.

The output power loss (in dB) variation with α of the light beam is the operating principle of the proposed multilayered sensor. The power loss variation is due to the modulation effects of the plasmonic structure coated onto a small length ‘L’ of the fiber (Figure 1). A laser diode mounted on a rotary stage is implemented as a source of monochromatic light. According to Snell’s law, ‘α’ and ‘θ’ (inside the fiber) variations are interrelated. The resonance condition (i.e., the matching of incident light and SPP wave-vectors) will be satisfied at a particular value of incident angle (i.e., α = α_SPR_). The transfer matrix method (TMM) is employed for the calculation of the normalized reflection coefficient (R) of p-polarized incident light [27]. The normalized output power after plasmonic modulation and taking Snell’s law into account can be calculated as:

(1) $$P\left(\alpha \right)=R{\left(\theta \right)}^{N_{ref}\left(\theta \right)}$$

In Equation (1), $N_{ref}\left(\theta \right)=L/\left(D\;tan\theta \right)$ represents the number of reflections corresponding to a light ray propagating at an angle ‘θ’ inside the fiber core. Here, D is the fiber core diameter. Finally, the power loss (PL) can be calculated as:

(2) $$PL\;\left(in\;dB\right)=10\;log_{10}\;\left(\frac{P_{ref}}{P_{out}}\right)$$

In the above equation, $P_{ref}$ is the normalized reference power and $P_{out}\;\left(=P\left(\alpha \right)\right)$ is the modulated normalized output power calculated from Equation (1).

**Figure 2:**
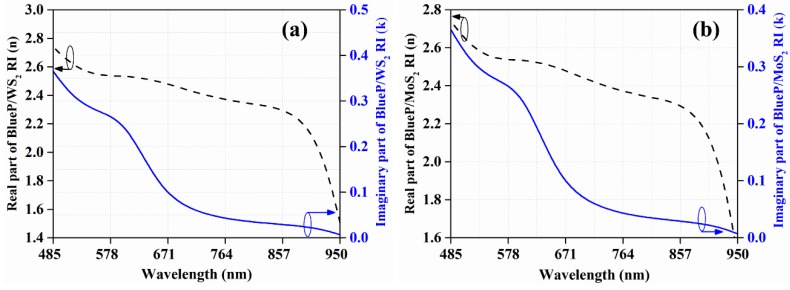
Spectral variation of the real part (n) and imaginary part (κ) of RI for monolayer (**a**) BlueP/WS_2_ heterostructure, and (**b**) BlueP/MoS_2_ heterostructure (this is the interpolated graph based on discrete data provided by ref [24]).

The fiber forming ability of HBL glasses [28] could be a remarkable development when applied in various photonic devices. In the NIR spectral region, HBL-based fiber has shown noteworthy optical properties, along with good mechanical and chemical stabilities [29,30]. The wavelength (λ)-dependent RI values of HBL are calculated from its dispersion relation [31], and the corresponding RI values of NaF are adapted from [32]. Furthermore, the clad material is coated with an Ag layer whose RI values are taken from [33]. The fourth layer is of a BlueP/TMD heterostructure with a thickness of 0.75 nm. This heterostructure will be in direct contact with the analyte, which will prevent Ag from possible degradation or oxidation issues. The thickness values of monolayer BlueP/WS_2_ and BlueP/MoS_2_ heterostructures are adapted, and the interpolated RI variations with wavelength are shown in Figure 2 [24]. 

The sensor design is aimed at the determination of malignancy in human liver tissues. The sensor is simulated with malignant metastatic tissue (‘MET’) liver tissue as an analyte and with normal (‘N’) liver tissue as a reference sample. The RI values of N and MET liver tissues are adapted from [34]. The work of Giannios et al. [34], which forms the basis for our simulation work, reported that liver tissue samples with 5 mm × 5 mm surface and 2 mm thickness can be prepared under a dissecting stereoscope. The work of Giannios et al. used the prism-coupling method to determine the RI of liver tissue samples. In the prism-coupling method, they ‘attached’ the liver tissue sample to the base of the glass prism without applying any intermediate liquid or external pressure in order to form a high-quality interface. It must be kept in mind that the above may be possible due to the high water content in liver tissue samples [35]. For that matter, another important difference between normal and malignant tissues is that the malignant tissue has significantly higher water content than the normal one. Against this backdrop, if we replicate the formation of high-quality interface involving liver tissues (as reported by Giannios et al.), the similar freshly excised liver tissue samples can also be seen to become attached to 2D heterostructures (see Figure 1) because most of the 2D heterostructure layers show sufficiently high binding with water molecules (via strong adsorption behavior towards O_2_ molecules). In this sequence, it is important to mention that a very recent study [36] quantitatively confirmed that the presence of TMDs on substrates resulted in an enhanced overall cellular morphology. Therefore, in view of the above points, the liver tissues can be attached to the 2D heterostructures in the proposed fiber SPR sensor. Figure of merit (FOM) is the measure of the overall performance of the fiber SPR sensor considering both of the following terms: (i) change in resonance angle ($\delta \alpha_{SPR}$) on small change in RI ($\delta n_{s}$) of MET with reference to N; and (ii) the angular width of PL spectrum (FWHM) of MET analyte. The FOM is defined as follows:

(3) $$FOM\;\left(in\;RIU^{-1}\right)=\frac{\delta \alpha_{SPR}}{\delta n_{s}}\;\times \;\frac{1}{FWHM}$$

The term $\delta \alpha_{SPR}/\delta n_{s}$ is considered to be the sensitivity of the system. The calculation of parameters and simulations are performed using MATLAB^®^ programming tool (Version 2016, Mathworks, Natick, MA, USA) while the field variation analysis is carried out with the COMSOL multiphysics tool (Version-v 5.20, COMSOL AB, Stockholm, STHLM, Sweden) (based on the finite element method).

## 3. Results and Discussion

In this section, the FOM enhancement is emphasized with the optimization of the coupled effect of λ and metal layer thickness (d_m_) for BlueP/WS_2_ and BlueP/MoS_2_ heterostructure-based fiber SPR sensors. The values of NaF and 2D heterostructure layers are fixed at 5 nm and 0.75 nm, respectively. 

### 3.1. Analysis of 2D (d_m_, λ) Variation of FOM for Sensor Designs with BlueP/WS_2_ & BlueP/MoS_2_ Heterostructures

**Figure 3:**
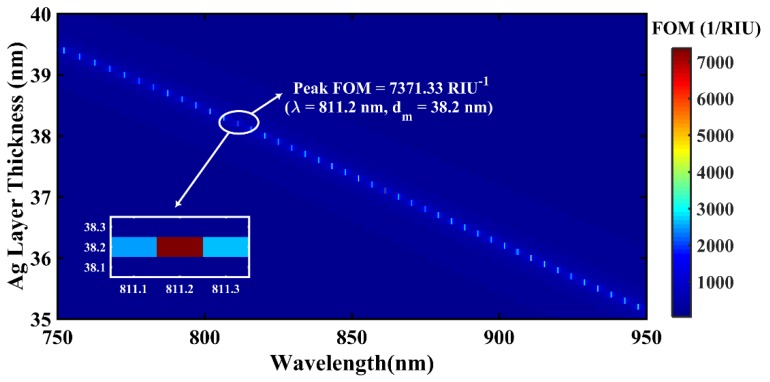
Simulated 2D (d_m_, λ) variation of FOM of a BlueP/WS_2_ heterostructure-based fiber SPR sensor. The magnified figure in the inset shows the maximum achieved FOM value for the optimized set of d_m_ and λ values.

Figure 3 illustrates the effect of simultaneous variation of Ag layer thickness (35–40 nm) and NIR wavelength (750–950 nm) on FOM in the case of a monolayer BlueP/WS_2_ heterostructure-based FOSPR sensor design. 

**Figure 4:**
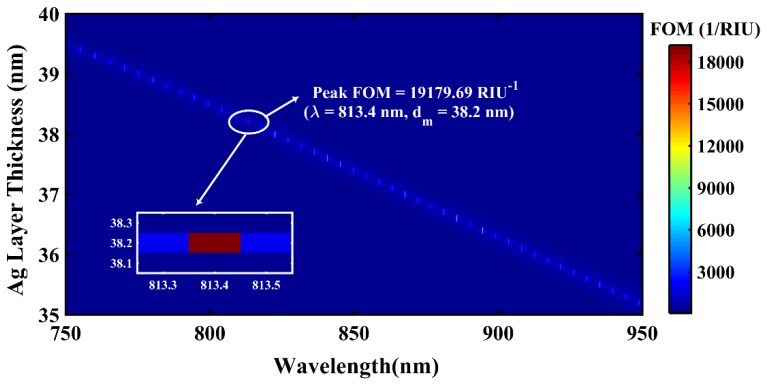
Simulated 2D (dm, λ) variation of FOM of a BlueP/MoS_2_ heterostructure-based fiber SPR sensor. The magnified figure in the inset shows the maximum achieved FOM value for the optimized set of d_m_ and λ values.

It is important to mention that the simulation was performed for a d_m_ range of 30–55 nm, but the prominent enhancement in FOM was observed in the range 35–40 nm only, as depicted in Figure 3. The above 2D plot shows that the FOM variation is highly sensitive to (d_m_, λ) combination with maximum FOM (M-FOM) of 7371.33 RIU^−1^ achieved at λ = 811.20 nm and d_m_ = 38.2 nm. This can be attributed to optimum radiation damping (ORD) for the corresponding SPR sensor structure. The M-FOM point is shown magnified in Figure 3. In this sequence, Figure 4 depicts the 2D variation of FOM for BlueP/MoS_2_ heterostructure-based sensor design. 

The M-FOM of BlueP/MoS_2_ heterostructure-based FOSPR sensor is 19179.69 RIU^−1^ for the combination λ = 813.4 nm and d_m_ = 38.2 nm. Interestingly, the d_m_ value corresponding to the M-FOMs of both the sensor designs is identical (i.e., 38.2 nm), and the λ-values are fairly close to each other (i.e., 811.2 nm for BlueP/WS_2_-based and 813.4 nm for BlueP/MoS_2_-based sensor designs). Despite identical d_m_ and very close values of λ, the M-FOM of BlueP/MoS_2_-based sensor (19179.69 RIU^−1^) is 2.6 times the M-FOM of BlueP/WS_2_-based sensor design (7371.33 RIU^−1^). This variation between the two M-FOM values could possibly be due to the reasonably different dispersion characteristics (i.e., spectral variation of n and κ) of BlueP/MoS_2_ and BlueP/WS_2_, as shown in Figure 2. A brief workflow of the proposed sensor can be summarized as follows: a tunable source and detector are the main components for the optical fiber sensor setup. An optically pumped laser can be utilized at λ = 811.20 nm [37] and a wavelength of 813.40 nm can be achieved by a Ti:Sapphire laser [38]. The shift in position of resonance angle can be observed at the detector side. The minimum possible angular shift that can be detected is 0.001° [39] Furthermore, the power loss (dB) measurement at resonance can be performed using an optical power meter, which can be used for calculation of power loss ratio (PLR).

### 3.2. Comparative Analysis between BlueP/WS_2_ and BlueP/MoS_2_ Heterostructure-Based Sensor Designs

**Figure 5:**
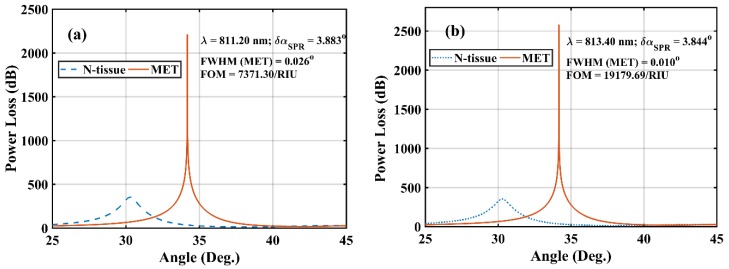
Simulated power loss (dB) variation with α (deg.) for N-MET analyte with d_m_ = 38.20 nm for proposed fiber SPR sensor with (**a**) BlueP/WS_2_ heterostructure and (**b**) BlueP/MoS_2_ heterostructure. The corresponding resonance condition and FWHM values are shown in the inset.

At this juncture, it is important to clearly understand the rationale behind the massive difference between M-FOM values as discussed in last section. In this context, Figure 5 represents the PL spectra (of N and MET tissues) for two sensor designs at their corresponding optimized λ values i.e., 811.2 nm (for BlueP/WS_2_) and 813.4 nm (for BlueP/MoS_2_). 

For MET tissue (i.e., analyte), the peak values of PL observed in the case of BlueP/WS_2_ and BlueP/MoS_2_ heterostructure-based sensors were 2213.062 dB and 2584.34 dB, respectively. Different peak PL values have a direct effect on the corresponding FWHM of the concerned PL spectra. The FWHM of PL spectrum is 0.026° (as shown in Figure 5a) and 0.010° (as shown in Figure 5b) for BlueP/WS_2_ and BlueP/MoS_2_ heterostructure-based sensors, respectively. For greater clarity, Table 1 lists the values of all concerned parameters corresponding to M-FOM (and the next highest FOM) achievable with the two sensor designs. The next prominent peaks show a comparative analysis with maximum FOM condition and provides another degree of freedom for experimental realization.

Table 1 clearly shows that the sensitivity of the two sensor designs under their ORD conditions (i.e., where M-FOM is achieved) is nearly identical (as shown in the shaded rows) and that is why a significantly greater M-FOM for BlueP/MoS_2_-based sensor is primarily due to much smaller FWHM (of MET tissue’s PL spectrum) compared with BlueP/WS_2_-based sensor. Evidently, the ORD condition leads to maximum light absorption at a specific set of concerned parameters, i.e., λ and d_m_. Consequently, the corresponding PL attains the highest magnitude, leading to minimum FWHM and maximum possible FOM. In other words, the greater the peak PL, the smaller the FWHM. This very point is also evident from the fact that BlueP/MoS_2_-based sensor possesses greater value of peak PL value and smaller FWHM leading to greater M-FOM than BlueP/WS_2_-based sensor. Another interesting point observable from Table 1 is that at the next prominent (λ, d_m_) combination, the M-FOM for the BlueP/MoS_2_ sensor design (i.e., 10177.50 RIU^−1^), is nearly half of the M-FOM corresponding to the ORD condition (i.e., 19179.69 RIU^−1^). Moreover, the sensitivity (183.1951°/RIU) is also smaller, and the optimal λ (i.e., 894.7 nm) is also considerably longer than the λ corresponding to the ORD condition (i.e., 813.4 nm). 

### 3.3. Field Analysis at Resonance Condition

It is important to appreciate that the interaction volume plays an important role in plasmonic sensor characteristics. Some of the research works based on fiber-optic SPR sensors have also demonstrated the field distribution along the analyte side under resonance conditions [40,41]. The field enhancement towards the analyte region increases the interaction volume. This field enhancement is a result of the maximum absorption of incident radiation at resonance, which denotes the field interaction with the sensing region. In other words, the field enhancement is corelated with the ORD condition, which, as discussed earlier, leads to maximum absorption. In that sense, the performance of heterostructure-based SPR sensor can be further explained in terms of the magnetic field enhancement under the corresponding ORD conditions.

**Figure 6:**
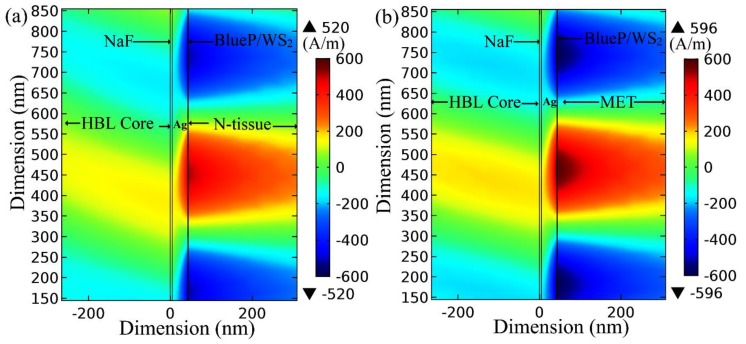
Magnetic field strength (A/m) at BlueP/WS_2_–analyte interface with λ = 811.20 nm, Ag = 38.2 nm, monolayer BlueP/WS_2_ thickness of 0.75 nm for (**a**) N-tissue as an analyte (RI = 1.3502 + 0.005i), and (**b**) MET as an analyte (RI = 1.3703 + 0.0032i).

The field analysis is performed using COMSOL Multiphysics tool (Version-v 5.20, COMSOL AB, Stockholm, STHLM, Sweden). Figure 6 shows the field variation of the BlueP/WS_2_ heterostructure-based sensor structure under the corresponding ORD conditions (λ = 811.20 nm and d_m_ = 38.2 nm) with N and MET tissues as the outermost media. The nature of the field distributions at resonance are in line with previously reported works on plasmonic sensors [40,41]. Higher electric field enhancement was observed for the Au-ITO-Au-coated fiber-optic SPR sensor than the Au-coated fiber-optic SPR sensor [40]. Furthermore, a magnetic field enhancement was reported at the ITO-Au-analyte interface in the case of fiber-optic SPR sensor designed for label-free and real-time monitoring of the IgG/anti-IgG biomolecular interaction [41].

Here, it is important to note that there is a significant change in the maximum value of field strength on changing the sensing medium from N-tissue (520 A/m) to MET tissue (596 A/m). Thus, there is approximately 14% field enhancement for MET tissue with reference to N-tissue. 

**Figure 7:**
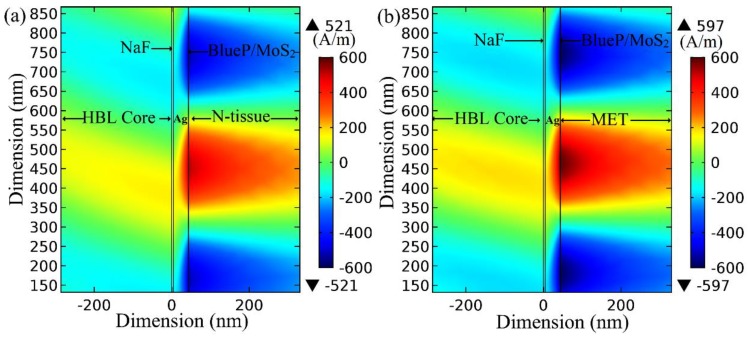
Magnetic field strength (A/m) at the BlueP/MoS_2_–analyte interface with λ = 813.40 nm, Ag = 38.20 nm, monolayer BlueP/MoS_2_ thickness of 0.75 nm for (**a**) N-tissue as an analyte (RI = 1.3501 + 0.005i), and (**b**) MET as an analyte (RI = 1.3703 + 0.0032i).

Furthermore, the field analysis was also performed for the BlueP/MoS_2_ heterostructure-based sensor structure under its corresponding ORD conditions (λ = 813.40 nm and d_m_ = 38.2 nm), as shown in Figure 7. 

Similar to Figure 6, the field enhancement factor is 1.14 in Figure 7 for MET tissue with reference to N tissue. Both of the above figures indicate a sufficiently large field variation when the analyte is taken as MET tissue with N tissue as a reference sample, leading to a very high practical sensitivity of the sensor. 

### 3.4. Comprehensive Performance Analysis under ORD Conditions

As discussed earlier, the FOM is an overall performance parameter, in consideration of the shift and width of analyte PL spectrum. Nonetheless, more comprehensive analysis can be carried in order to understand the simulated sensor model in as close to a practical realization as possible. In this regard, it is important to mention that:

(i) the wavelength of sensor operation should ideally be as large as possible in the NIR range, owing to the lower photodamage of the analyte (biosamples, in particular) [42] and the smaller Rayleigh scattering factor, RSF (i.e., λ^−4^),
(ii) the ratio (PLR) of peak PL (MET tissue) to peak PL (N tissue) under corresponding ORD conditions (as depicted in Figure 5) can be added as another performance element,
(iii) as discussed in Section 3.3, field enhancement factor (FEF) is another possible inclusion to the sensor’s performance evaluation.

Of course, PLR and FEF should be as large and RSF should be as small as possible (within practical limits). At this stage, a combined figure of merit (C-FOM) of the sensor as a union of M-FOM, RSF, FEF, and corresponding PLR can be envisaged.

(4) $$CFOM=\frac{MFOM\times PLR\times FEF}{RSF}$$

Table 2 lists the values of required parameters leading to calculation of C-FOM (in µm^4^/RIU as per Equation (4)).

Table 2 clearly indicates that the BlueP/MoS_2_-based sensor design provides a superior overall performance due not only to its greater M-FOM, but also to its slightly longer ORD wavelength (leading to smaller RSF) and greater PLR (as is also visible in Figure 5). The BlueP/WS_2_-based sensor design may not be preferred due to smaller magnitudes of M-FOM and PLR, even though the FEF and RSF are almost identical. Hence, specific biosensing applications that require as little signal scattering and photodamage as possible, along with a more prominent PL spectrum, should be carried out with the BlueP/MoS_2_ heterostructure-based sensor design. The most prominent M-FOM values are also compared with some other research works as shown in Table 3, which points towards the significantly enhanced sensing performance achievable with the proposed sensor designs.

Finally, it is necessary to discuss a few possible limitations of the proposed sensor design. For instance, the sensor design has a very delicate dependence on the light wavelength, which has to be fine-tuned very carefully in order to reach the desired performance enhancement. If the wavelength drifts by some amount, the envisioned performance enhancement may also drop accordingly. Furthermore, the data for simulation of the proposed sensor adapted from Giannios et al. [34] is not specific to bacterial phases (e.g., lag, exponential, stationary, and death phases). Nevertheless, the above work reports that the cell morphology of the tissues utilized in their measurements did not change with time. Therefore, the simulation results and the conclusions of the proposed study may vary if there is any variation (for any reason) in the bacterial phase of the tissue. Also, the tuning of angle (α) should be performed carefully and with as fine a resolution as possible (e.g., 0.001° [39]).

## 4. Conclusions

The application of a 2D heterostructure as the analyte interacting layer in a fluoride fiber SPR sensor aimed at detecting malignant liver tissue (MET, with reference to normal tissue N) was explored in this work. The incident wavelength (λ) and metal layer thickness (d_m_) were optimized in order to achieve the maximum figure of merit (M-FOM). Large M-FOM values of 7371.30 RIU^−1^ and 19179.69 RIU^−1^ were obtained for BlueP/WS_2_ and BlueP/MoS_2_ heterostructure-based sensor designs, respectively. The simulation results were discussed in terms of optimum radiation damping (ORD) occurring at the metal–heterostructure–analyte interfaces. In this context, the field strength at resonance was also simulated for the sensor designs and discussed in line with ORD. Making the analysis more comprehensive, the sensors’ performances were evaluated in terms of a combined parameter (C-FOM) defined under ORD conditions as a union of M-FOM, peak power loss ratio, Rayleigh scattering factor, and field enhancement factor. The comprehensive analysis indicates that with C-FOM of 70046.01 µm^4^/RIU, the BlueP/MoS_2_ heterostructure-based sensor design was capable of providing massively superior overall sensing performance compared with BlueP/WS_2_ heterostructure-based sensor design (C-FOM = 22871.63 µm^4^/RIU).

## Figures and Tables

**Table 1:** Calculated values of constituent parameters leading to M-FOM (shaded rows) and the next highest FOM values.

S.N.	Monolayer 2D Heterostructure	d_m_ (nm)	λ (nm)	δn_s_ (RIU)	δα_SPR_ (°)	Sensitivity (°/RIU)	FWHM (°)	M-FOM (RIU^−1^)
1	BlueP/WS_2_	38.2	811.2	0.02026040	3.883	191.6547	0.026	7371.30
2	BlueP/WS_2_	37.1	861.3	0.02004785	3.727	185.9052	0.026	7150.19
3	BlueP/MoS_2_	38.2	813.4	0.02004203	3.844	191.7969	0.010	19,179.69
4	BlueP/MoS_2_	36.4	894.7	0.01991865	3.649	183.1951	0.018	10,177.50

**Table 2:** Combined performance parameter (C-FOM) for two different sensor designs.

Heterostructure	M-FOM (RIU^−1^)	λ_ORD_ (µm)	RSF (µm^−4^)	PLR	FEF	C-FOM (µm^4^/RIU)
BlueP/MoS_2_	19,179.69	0.8134	2.284	7.317	1.14	70,046.01
BlueP/WS_2_	7371.30	0.8112	2.301	6.263	1.14	22,871.63

**Table 3:** FOM comparison of proposed scheme with some of the research works.

References	SPR Modalities	FOM (RIU^−1^)
Sharma and Kaur [22]	Samarium-doped fiber coated with 2D material	6904.012 (graphene) 5897.082 (MoS_2_)
Sharma and Kaur [43]	Chalcogenide fiber sensor with polymer and 2D layer	1647 (λ = 1200 nm)
Popescu et al. [44]	Bragg fiber with AS_2_S_3_ chalcogenide layer	233.10
Feng et al. [45]	Long range SPR on side-polished fiber with MgF_2_ layer	156.19
Bialiayeu et al. [46]	Tilted fiber Bragg gratings coated with silver nanowire	3700
Gazzaz and Berini [47]	Waveguide Bragg gratings supporting surface plasmons	1000
This work	Fluoride fiber coated with Ag layer (38.2 nm) and (BlueP/TMDs) heterostructure	19,179.69 (BlueP/MoS_2_) 7371.30 (BlueP/WS_2_)

## References

[1] Balendhran S., Walia S., Nili H., Sriram S., Bhaskaran M. (2015). Elemental analogues of graphene: Silicene, germanene, stanene, and phosphorene. Small.

[2] Rubio-Bollinger G., Guerrero R., de Lara D., Quereda J., Vaquero-Garzon L., Agraït N., Bratschitsch R., Castellanos-Gomez A. (2015). Enhanced Visibility of MoS_2_, MoSe_2_, WSe_2_ and Black-Phosphorus: Making Optical Identification of 2D Semiconductors Easier. Electronics.

[3] Mas-Ballesté R., Gómez-Navarro C., Gómez-Herrero J., Zamora F. (2011). 2D materials: To graphene and beyond. Nanoscale.

[4] Song B., Li D., Qi W., Elstner M., Fan C., Fang H. (2010). Graphene on Au(111): A highly conductive material with excellent adsorption properties for high-resolution bio/nanodetection and identification. ChemPhysChem.

[5] Koppens F.H.L., Chang D.E., de Abajo F.J. (2011). Graphene Plasmonics: A Platform for Strong Light-Matter Interactions. Nano Lett..

[6] Bonaccorso F., Sun Z., Hasan T., Ferrari A.C. (2010). Graphene Photonics and Optoelectronics. Nat. Photonics.

[7] Wu L., Guo J., Wang Q., Lu S., Dai X., Xiang Y., Fan D. (2017). Sensitivity enhancement by using few-layer black phosphorus-graphene/TMDCs heterostructure in surface plasmon resonance biochemical sensor. Sens. Actuators B Chem..

[8] Novoselov K.S., Mishchenko A., Carvalho A., Neto A.H.C. (2016). 2D materials and van der Waals heterostructures. Research.

[9] Mcgaughey G.B., Gagne M., Rappe A.K. (1998). π-Stacking Interactions. J. Biol. Chem..

[10] Kong L., Enders A., Rahman T.S., Dowben P. (2014). A Molecular adsorption on graphene. J. Phys. Condens. Matter.

[11] Zhao Y., Li X., Zhou X., Zhang Y. (2016). Review on the graphene based optical fiber chemical and biological sensors. Sens. Actuators B Chem..

[12] Maharana P.K., Srivastava T., Jha R. (2014). On the Performance of Highly Sensitive and Accurate Graphene-on-Aluminum and Silicon-Based SPR Biosensor for Visible and Near Infrared. Plasmonics.

[13] Pandey A.K., Sharma A.K. (2018). Simulation and analysis of plasmonic sensor in NIR with fluoride glass and graphene layer. Photonics Nanostruct. Fundam. Appl..

[14] Wu L., Guo J., Dai X., Xiang Y., Fan D. (2017). Sensitivity Enhanced by MoS2-Graphene Hybrid Structure in Guided-Wave Surface Plasmon Resonance Biosensor. Plasmonics.

[15] Wu L., Jia Y., Jiang L., Guo J., Dai X., Xiang Y., Fan D. (2017). Sensitivity Improved SPR Biosensor Based on the MoS2/Graphene–Aluminum Hybrid Structure. J. Light. Technol..

[16] Maurya J.B., Prajapati Y.K., Singh V., Saini J.P., Tripathi R. (2016). Improved performance of the surface plasmon resonance biosensor based on graphene or MoS_2_ using silicon. Opt. Commun..

[17] Sharma A.K., Pandey A.K. (2018). Blue Phosphorene/MoS2 Heterostructure based SPR Sensor with Enhanced Sensitivity. IEEE Photonics Technol. Lett..

[18] Liang G., Luo Z., Liu K., Wang Y., Dai J., Duan Y. (2016). Fiber Optic Surface Plasmon Resonance–Based Biosensor Technique: Fabrication, Advancement, and Application. Crit. Rev. Anal. Chem..

[19] Sharma A.K., Pandey A.K., Kaur B. (2018). A Review of advancements (2007–2017) in plasmonics-based optical fiber sensors. Opt. Fiber Technol..

[20] Fu H., Zhang S., Chen H., Weng J. (2015). Graphene Enhances the Sensitivity of Fiber-Optic Surface Plasmon Resonance Biosensor. IEEE Sens. J..

[21] Mishra A.K., Mishra S.K., Verma R.K. (2016). Graphene and Beyond Graphene MoS2: A New Window in Surface-Plasmon-Resonance-Based Fiber Optic Sensing. J. Phys. Chem. C.

[22] Sharma A.K., Kaur B. (2018). Fiber optic SPR sensing enhancement in NIR via optimum radiation damping catalyzed by 2D materials. IEEE Photonics Technol. Lett..

[23] Sharma A.K., Kaur B. (2019). Simulation of multilayered heterojunction-based chalcogenide fiber SPR sensor with ultrahigh figure of merit in near infrared. IEEE Sens. J..

[24] Peng Q., Wang Z., Sa B., Wu B., Sun Z. (2016). Electronic structures and enhanced optical properties of blue phosphorene/transition metal dichalcogenides van der Waals heterostructures. Sci. Rep..

[25] Jariwala D., Davoyan A.R., Tagliabue G., Sherrott M.C., Wong J., Atwater H.A. (2016). Near-Unity Absorption in van der Waals Semiconductors for Ultrathin Optoelectronics. Nano Lett..

[26] ZBLAN Fluoride Glass Fibers & Cables. https://www.fiberlabs.com/fiberindex/fiber-stock.

[27] Sharma A.K. (2013). Plasmonic biosensor for detection of hemoglobin concentration in human blood: Design considerations. J. Appl. Phys..

[28] Bendow B., Brown R.N., Lipson H.G., Drexhage M.G., Moynihan C.T. (1982). Infrared edge absorption in fluorohafnate glass. Appl. Opt..

[29] Tran D.C., Sigel G.H., Bendow B. (1984). Heavy Metal Fluoride Glasses and Fibers: A Review. J. Lightw. Technol..

[30] Pandey A.K., Sharma A.K., Basu R. (2017). Fluoride Glass based Surface Plasmon Resonance Sensor in Infrared region: Performance Evaluation. J. Phys. D Appl. Phys..

[31] Ghatak A., Thyagarajan K. (1998). An Introduction to Fiber Optics.

[32] Li H.H. (1980). Refractive index of alkaline earth halids and its wavelength and temperature derivatives. J. Phys. Chem..

[33] Rakic A.D., Djurisic A.B., Elazar J.M., Majewski M.L. (1998). Optical properties of metallic films for vertical-cavity optoelectronic devices. Appl. Opt..

[34] Giannios P., Toutouzas K.G., Matiatou M., Stasinos K., Konstadoulakis M.M., Zografos G.C., Moutzouris K. (2016). Visible to near-infrared refractive properties of freshly-excised human-liver tissues: Marking hepatic malignancies. Sci. Rep..

[35] Ross K.F.A., Gordon R.E. (1982). Water in malignant tissue, measured by cell refractometry and nuclear magnetic resonance. J. Microsc..

[36] Palumbo A., Tourlomousis F., Chang R.C., Yang E.-H. (2018). Influence of Transition Metal Dichalcogenide Surfaces on Cellular Morphology and Adhesion. ACS Appl. Biol. Mater..

[37] Wolfram T., Vojak B.A., Edward T., Maas J., Burnham R.D. (1990). Optically Pumped Laser. U.S. Patent.

[38] Lattice Trap Improves Optical Clocks. http://optics.org/article/22237.

[39] Mao Y., Bao Y., Wang W., Li Z., Li F., Niu L. (2011). Development and Application of Time-Resolved Surface Plasmon Resonance Spectrometer. Am. J. Anal. Chem..

[40] Li L., Zhang X., Liang Y., Guang J., Peng W. (2016). Dual-channel fiber surface plasmon resonance biological sensor based on a hybrid interrogation of intensity and wavelength modulation. J. Biomed. Opt..

[41] Li L., Liang Y., Guang J., Cui W., Zhang X., Masson J.-F., Peng W. (2017). Dual Kretschmann and Otto configuration fiber surface plasmon resonance biosensor. Opt. Express.

[42] Ziblat R., Lirtsman V., Davidov D., Aroeti B. (2006). Infrared surface plasmon resonance: A novel tool for real time sensing of variations in living cells. Biophys. J..

[43] Sharma A.K., Kaur B. (2018). Optical Fiber Technology Chalcogenide fiber-optic SPR chemical sensor with MoS 2 monolayer, polymer clad, and polythiophene layer in NIR using selective ray launching. Opt. Fiber Technol..

[44] Popescu V., Puscas N., Perrone G. (2018). The Sensing Characteristics of a Bragg Fiber Based Plasmonic Biosensor Using an As2S3 Chalcogenide Layer. Optics.

[45] Xia K., Feng X., Yang M., Luo Y., Tang J., Guan H., Lu H., Yu J., Zhang J., Chen Y. (2017). Long range surface plasmon resonance sensor based on side polished fiber with a buffer layer of magnesium fluoride. Opt. Quant. Electron..

[46] Bialiayeu A., Bottomley A., Prezgot D., Ianoul A., Albert J. (2012). Plasmon-enhanced refractometry using silver nanowire coatings on tilted fibre Bragg gratings. Nanotechnology.

[47] Gazzaz K., Berini P. (2015). Theoretical biosensing performance of surface plasmon polariton Bragg gratings. Appl. Opt..

